# Current Approach and Predictors of Clinical Outcomes in Adults With Spinal Ependymomas

**DOI:** 10.7759/cureus.89320

**Published:** 2025-08-04

**Authors:** Alan Hernández-Hernández, German López-Valencia, Marco Antonio Muñuzuri-Camacho, Rodrigo Uribe-Pacheco, Tomas Moncada-Habib, Eliezer Villanueva-Castro, Ana Laura Calderón-Garcidueñas, Itzel Ariadna Dehesa-Hernandez, Juan Antonio Ponce-Gómez, Juan Nicasio Arriada-Mendicoa

**Affiliations:** 1 Spine Surgery, National Institute of Neurology and Neurosurgery "Manuel Velasco Suárez", Mexico City, MEX; 2 Neurosurgery, National Institute of Neurology and Neurosurgery "Manuel Velasco Suárez", Mexico City, MEX; 3 Neurosurgery, Neuro Center, Cancer Center Tec100, Santiago de Querétaro, MEX; 4 Neurosurgery, University of Arkansas for Medical Sciences, Little Rock, USA; 5 Neuropathology, National Institute of Neurology and Neurosurgery "Manuel Velasco Suárez", Mexico City, MEX; 6 Radiology, National Institute of Rehabilitation, Mexico City, MEX

**Keywords:** disease-free survival, ependymoma, magnetic resonance imaging, neurosurgical procedures, spinal neoplasms

## Abstract

Background

Spinal ependymomas are the most common intradural tumors in adults and frequently lead to progressive neurological decline due to spinal cord compression. They typically present with subacute symptoms. The 2016 WHO classification stratifies them by histological grade, with recent updates incorporating molecular features. Contrast-enhanced MRI remains the gold standard for diagnosis. Surgical resection is the mainstay of treatment, with gross total resection (GTR) linked to superior clinical and oncological outcomes.

Methods

We conducted a retrospective analysis of adult patients who underwent surgery for spinal ependymoma between 2010 and 2023. Demographic, clinical, radiological, and histopathological data were collected. Functional status was evaluated using the McCormick, Sourse, and modified Rankin scales. Statistical analyses included Cox proportional hazards models and Kaplan-Meier survival estimates.

Results

Fifty-six patients were included (mean age: 36.8 years; 54.5% male). Tumors were most commonly located in the cervical (54.5%) and thoracic (52.7%) regions. The predominant symptoms were sensory disturbances (92.7%), motor weakness (90.9%), and pain (78.2%). GTR was achieved in 63.6% of cases. Overall, 89.1% of patients experienced functional improvement, with motor recovery observed in 76.4%. Progression-free survival (PFS) at 28 months was 62.1%, significantly higher in patients with GTR (82.6%) compared to subtotal resection (41.9%). Thoracic tumor location was associated with poorer functional recovery (HR: 4.7, p = 0.022), while GTR significantly decreased recurrence risk (HR: 0.25, p = 0.028).

Conclusions

Spinal ependymomas predominantly affect adults in their fourth decade, with a slight male predominance. GTR significantly improves PFS and reduces the likelihood of recurrence, underscoring its therapeutic value. Thoracic location and the presence of syringomyelia are adverse prognostic factors. Optimal outcomes require an aggressive but individualized surgical strategy, tailored to tumor location and preoperative functional status.

## Introduction

Spinal ependymomas are intramedullary tumors with variable clinical presentations, often leading to significant neurological deficits in adults. Due to their potential for aggressive behavior, timely diagnosis and treatment are essential to achieve favorable outcomes. However, clinical data on prognostic factors influencing both functional recovery and progression-free survival (PFS) in adult patients with spinal ependymomas remain limited, particularly in underrepresented populations such as those in Latin America.

Although retrospective in design, this study was guided by the hypothesis that specific clinical and radiological features - such as tumor location, extent of resection, and the presence of syringomyelia - are associated with postoperative functional outcomes and disease progression.

The aim of this study is to characterize the clinical, radiological, and pathological features of spinal ependymomas in adult patients with histologically confirmed tumors who underwent surgical resection at a national referral center and to identify predictors of both functional recovery and PFS, with the goal of supporting neurosurgical decision-making regarding extent of resection and follow-up planning based on individual risk.

Given the scarcity of region-specific data and limited integration of functional scales in prior studies, this analysis aims to enhance risk stratification and support evidence-based treatment planning.

## Materials and methods

Study design and patients

This was a retrospective observational study based on prospectively maintained data from a single national tertiary referral center. The study cohort included adult patients with histologically confirmed spinal ependymomas who underwent surgical resection between January 2010 and December 2023 at the National Institute of Neurology and Neurosurgery in Mexico City, Mexico. This institution serves as a national reference center for complex spinal pathologies and maintains a dedicated spine surgery database that is routinely updated with demographic, clinical, radiological, and surgical information.

Inclusion criteria were as follows: (1) age ≥18 years; (2) histopathological confirmation of spinal ependymoma; and (3) complete preoperative and postoperative data available in the institutional electronic registry. Exclusion criteria included (a) non-ependymoma histology; (b) incomplete clinical or imaging data; and (c) follow-up period shorter than six months.

Surgical technique

All patients underwent a standardized posterior midline approach under general anesthesia. In the prone position on a radiolucent table, head fixation was achieved with a memory foam mask or Mayfield clamp for cervical lesions. Intraoperative neuromonitoring (motor and somatosensory evoked potentials) was used in all cases to minimize neurological risk.

A midline skin incision extended one vertebral level above and below the tumor margins. Subperiosteal dissection exposed the laminae, followed by laminectomies to ensure full cranio-caudal visualization based on preoperative MRI. A longitudinal durotomy was performed under microscopy, extending beyond both poles of the tumor.

The tumor-cord interface was identified along gliotic planes when present. En bloc resection was the objective; internal debulking was minimized to avoid traction injury. High magnification allowed continuous assessment of the tumor-cord boundary to preserve functional tissue. Following resection, the cavity was irrigated, and meticulous hemostasis was ensured.

Dural closure was watertight using 6-0 Prolene with or without patch augmentation. Laminoplasty was performed in all cases using titanium miniplates and screws to preserve stability. Standard layered closure was completed.

Patients were monitored postoperatively in the recovery unit and then transferred to the neurosurgical ward. Neurological examinations were performed regularly, and contrast-enhanced MRI was obtained within 72 hours to evaluate the extent of resection and detect early complications.

Outcome assessment and follow-up

Initial outpatient evaluation occurred 10-15 days after surgery, including wound inspection, suture removal, and neurological examination. Follow-ups were scheduled at 3, 6, and 12 months and then annually as needed. Each visit included a functional evaluation focusing on motor strength, sensory deficits, sphincter control, and gait.

Functional status was graded using the Modified McCormick Scale (MMCS) [1], the Modified Rankin Scale (mRS) [2], and the Sourse Score (SS) [3] - a recently validated three-point tool specifically designed to predict outcomes after spinal ependymoma resection by integrating motor, sensory, and sphincter deficits. These three scales were selected to provide a multidimensional assessment: MMCS for spinal cord functional grading, mRS for global disability, and SS for prognostic stratification. This approach ensured clinical and predictive relevance in our cohort.

While both the MMCS and mRS assess functional status, they emphasize different aspects: the MMCS is specifically designed for spinal cord disorders and reflects neurological deficits, particularly in motor and gait function, whereas the mRS is a global disability scale originally developed for stroke patients, which incorporates broader aspects of independence in daily life. These differences may explain discordant grading in some cases.

Postoperative MRIs were obtained within one to three months to correlate radiological and clinical status and assess for adjuvant treatment indications. Complications such as cerebrospinal fluid (CSF) fistulas or meningitis were treated with lumbar drainage and antibiotics in collaboration with the infectious disease service.

Final outcome data were based on the most recent clinical documentation and radiological studies available. Functional assessments were independently performed by two attending neurosurgeons with expertise in spinal oncology. Discrepancies in scale grading were resolved through discussion and consensus. No blinding was applied during clinical evaluations.

Data collection and definitions

Demographic, clinical, imaging, and histopathological data were extracted from electronic medical records using a standardized protocol. Two attending neurosurgeons with expertise in spinal oncology were responsible for data abstraction. When discrepancies arose during chart review, these were resolved through joint discussion and consensus. No formal blinding was implemented at any stage of data collection or analysis.

Collected variables included age, sex, tumor histology and grade, spinal level, tumor volume, presence of associated cysts or syringomyelia, and neurological syndromes. Clinical presentation was documented pre- and postoperatively. Functional outcomes were assessed using the MMCS, the SS, and the mRS.

Gross total resection (GTR) was defined as the absence of radiological evidence of residual tumor on early postoperative contrast-enhanced MRI, as assessed by both a spine neurosurgeon and a neuroradiologist. Imaging studies were reviewed independently, with discrepancies resolved by consensus.

Cases with missing critical variables - such as histopathology, imaging, or follow-up documentation - were excluded from the final analysis, as detailed in the patient flow diagram. No imputation methods were employed for missing data.

Statistical analysis

Statistical analyses were conducted using Statistical Product and Service Solutions (SPSS, version 29; IBM SPSS Statistics for Windows, Armonk, NY). Descriptive statistics included means, medians, standard deviations, and interquartile ranges. Associations between variables were explored using Spearman’s correlation (⍴). Predictive factors for unfavorable outcomes and recurrence were evaluated using Cox proportional hazards models. Functional scales were dichotomized:

MMCS: favorable (grades 1-2), unfavorable (grades 3-5)

SS: favorable (0-1), unfavorable (2-3)

mRS: favorable (0-2), unfavorable (3-6)

Kaplan-Meier survival analysis was used to compare PFS across subgroups. A p-value < 0.05 and 95% confidence intervals were considered statistically significant.

Ethical considerations

This study was approved by the Ethics and Research Committee of the National Institute of Neurology and Neurosurgery “Manuel Velasco Suárez” (approval number: 003/25). Due to its retrospective, non-interventional nature, informed consent for study participation was waived. Nevertheless, all patients and their legal representatives provided informed consent for surgical treatment and clinical care.

## Results

A total of 74 patients were initially evaluated for inclusion. Eighteen were excluded due to unavailable imaging data (n = 9, 50%), histopathological reclassification (n = 5, 27.8%), or incomplete clinical information (n = 4, 22.2%). Thus, 56 patients met all inclusion criteria and comprised the final study cohort. The mean age at diagnosis was 36.82 years (SD: 12.7), with a slight male predominance (30 males, 54.5%).

**Figure 1:**
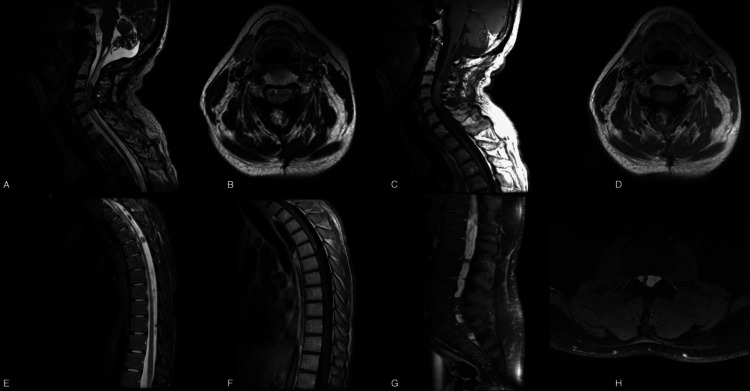
Magnetic resonance imaging of spinal ependymomas A–D. Cervical ependymoma (C3–C5): Lesion with a centripetal location exhibiting a predominantly solid, heterogeneous appearance. A non-recent hemorrhagic component is evident at the caudal pole, and a cystic component is present rostrally. The tumor causes spindle-shaped spinal cord expansion and is associated with edema extending from the bulbomedullary junction to C7. (A, B) Sagittal and axial T1-weighted images show the solid component as heterogeneously hypointense. (C, D) Sagittal and axial T1-weighted images with gadolinium contrast reveal homogeneous enhancement. Histopathology: Classic ependymoma, WHO grade 2. E–F. Cervicodorsal ependymoma: Spindle-shaped solid tumor involving the cervicodorsal region. (E) Sagittal T2-weighted image demonstrates dilation of the central canal. (F) Sagittal T1-weighted image shows the tumor is isointense relative to the spinal cord. Histopathology: Classic ependymoma, WHO grade 2. G–H. Myxopapillary ependymoma with dissemination: Multiple intradural lesions located in the cauda equina and sacral region (S2–S3), causing distortion of nerve roots. Lesions are solid, fusiform, variable in size, and well demarcated. (G) Sagittal and (H) axial gadolinium-enhanced STIR images demonstrate avid, heterogeneous enhancement. Histopathology: Myxopapillary ependymoma, WHO grade 2.

**Figure 2:**
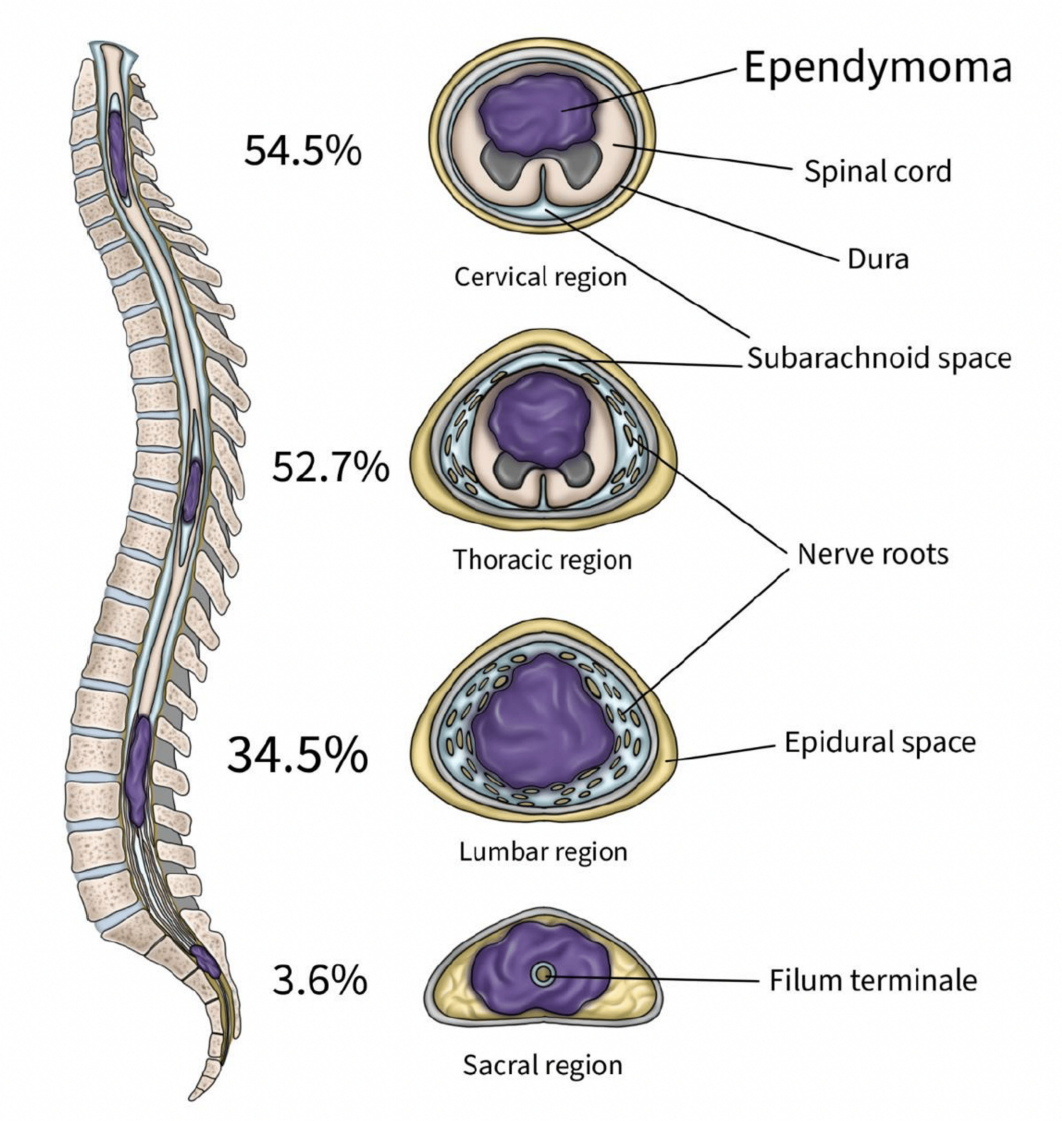
Distribution of spinal ependymoma locations by the spinal segment in our case series Illustration depicting the spinal cord segmented by anatomical regions, highlighting the frequency of spinal ependymoma occurrence per segment based on our cohort (n = 56). Percentages indicate the proportion of tumors found at each spinal level. Cervical and thoracic segments show the highest incidence, consistent with clinical presentation patterns in adult patients. Illustration created by author AHH.

Tumors were predominantly located in the cervical (54.5%) and thoracic (52.7%) spinal regions. Several patients presented with lesions spanning multiple contiguous levels, such as cervicothoracic or thoracolumbar tumors, which explains why percentages exceed 100%. The anatomical distribution of tumors by spinal segment is summarized in Figure 2. Representative MRI features of selected cases are shown in Figure 1.

Clinical presentations were heterogeneous, including pain (78.2%), sensory disturbances (92.7%), motor weakness (90.9%), spasticity (21.8%), urinary incontinence (36.4%), and bowel dysfunction (30.9%) (see Table 1 for full clinical breakdown). The median follow-up was 17 months (IQR: 31).

Spinal cord syndrome presentations varied: 41.8% of patients showed no spinal cord signs, while 32.7% exhibited a complete syndrome. Preoperative functional grading revealed grade 3 as the most frequent on both the MMCS and SS. The mRS was more evenly distributed, with grades 3 and 5 being the most prevalent (23.6% each) (see Table 1). The mean tumor volume was 7.27 ± 9.62 cc, reflecting disease heterogeneity.

**Table 1:** Preoperative clinical, demographic, and neuroimaging characteristics of adult patients with spinal ependymoma (n = 56) Demographic, clinical, and neuroimaging characteristics of 56 adult patients diagnosed with spinal ependymoma prior to surgery. Data are represented as mean ± standard deviation (SD) for continuous variables, or as number (percentage) for categorical variables. Functional status was assessed using the McCormick, Sourse, and modified Rankin scales. Syringomyelia data correspond to a subset of 41 patients with complete imaging records. Note: Percentages in the “Tumor localization” section may exceed 100% because some tumors extended across multiple spinal regions (e.g., cervicothoracic).

Variable	Value
Age at diagnosis (years)	36.82 ± 12.74
Male	30 (54.5%)
Female	25 (45.5%)
Number of spinal levels involved	4.20 ± 2.09
Tumor localization, n (%)	
Cervical	30 (54.5%)
Thoracic	29 (52.7%)
Lumbar	19 (34.5%)
Sacral	2 (3.6%)
Clinical presentation, n (%)	
Pain	43 (78.2%)
Sensory disturbance	51 (92.7%)
Spasticity	12 (21.8%)
Weakness	50 (90.9%)
Urinary incontinence	20 (36.4%)
Bowel dysfunction	17 (30.9%)
Urinary symptoms	19 (34.5%)
Fecal incontinence	14 (25.5%)
Medullar syndrome, n (%)	
None	23 (41.8%)
Anterior	2 (3.6%)
Posterior	2 (3.6%)
Central	5 (9.1%)
Cone	0 (0.0%)
Cauda equina	5 (9.1%)
Hemisection	0 (0.0%)
Complete	18 (32.7%)
Tumor volume (cc)	7.27 ± 9.62
Syringomyelia, n (%) (n=41)	19 (34.5%)
Functional scales, n (%)	
McCormick (%)	
Grade 1	1 (1.8%)
Grade 2	16 (29.1%)
Grade 3	18 (32.7%)
Grade 4	14 (25.5%)
Grade 5	6 (10.9%)
Sourse Score (%)	
Grade 1	4 (7.3%)
Grade 2	19 (34.5%)
Grade 3	32 (58.2%)
Modified Rankin Scale (mRS) (%)	
Grade 1	7 (12.7%)
Grade 2	11 (20.0%)
Grade 3	13 (23.6%)
Grade 4	11 (20.0%)
Grade 5	13 (23.6%)

Among patients with complete neuroimaging data (n = 41), 53.6% had syringomyelia, which correlated significantly with cervical (R = 0.51, p = 0.001) and lumbar (R = -0.68, p < 0.001) tumor locations, and with recurrence (R = 0.31, p = 0.043).

Surgical data showed that 36.4% of patients underwent subtotal resection, while 63.6% achieved GTR. The mean number of surgeries per patient was 1.16, indicating that some required reoperations due to recurrence or progression.

**Figure 3:**
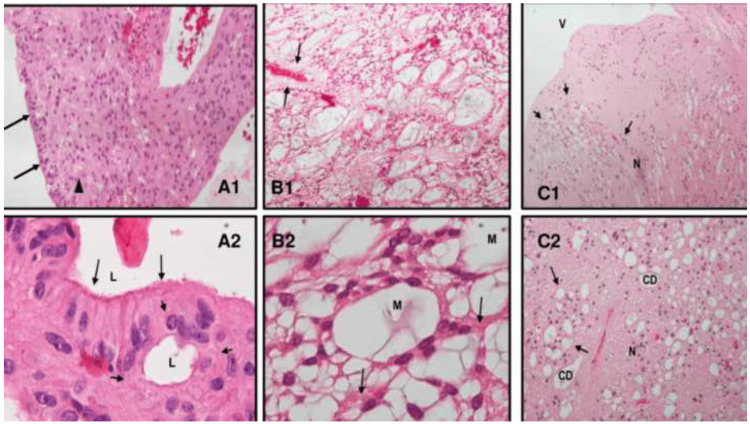
Histopathological features of spinal ependymoma subtypes (hematoxylin & eosin staining) A1–A2. Classic ependymoma (WHO Grade 2): A1 shows compact fibrillary perivascular pseudorosettes (arrowhead) and a large true rosette composed of elongated tumor cells with fibrillary cytoplasm and vesicular nuclei (long arrows). A2 is a higher magnification detail highlighting two true rosettes: one large (long arrows) and one small (short arrows). Magnifications: 50× (A1) and 400× (A2). B1–B2. Myxopapillary ependymoma (WHO Grade 2): B1 demonstrates a radial arrangement of tumor cells around vascularized fibromyxoid cores (arrows). B2 shows tumor cells with fibrillary cytoplasm (arrows) and microcysts containing myxoid material (M). Magnifications: 50× (B1) and 400× (B2). C1–C2. Subependymoma (WHO Grade 1): C1 illustrates tumor protrusion into the ventricle (V) with clusters of nuclei (N) within an abundant fibrillary background forming nodules (arrows) with focal microcystic changes. C2 highlights clustered nuclei (N) and focal microcystic changes (arrows). Magnifications: 50× (C1) and 400× (C2).

Histopathologically, classic ependymomas were the most common subtype (52.7%), followed by myxopapillary (29.1%), anaplastic (12.7%), and subependymomas (5.4%). According to WHO grading, most tumors were classified as grade 2 (81.8%), with fewer cases identified as grade 3 (12.7%) or grade 1 (5.5%). Representative histopathological features of each ependymoma subtype are illustrated in Figure 3, including perivascular pseudorosettes, myxoid microcysts, and subependymal nodularity.

Clinical improvement was observed in 89.1% of patients, with symptom-specific recovery most notable for motor weakness (76.4%), followed by pain (40.0%) and sensory disturbances (32.7%). Lower improvement rates were seen in autonomic symptoms, including urinary (16.4%), bowel dysfunction (10.9%), and spasticity (5.5%).

Adjuvant treatments consisted primarily of fractionated radiotherapy (32.7%), while chemotherapy was rare (1.8%). No cases of metastatic spread were documented.

Postoperative complications were infrequent, each occurring in fewer than 6% of patients. These included hemodynamic instability, mechanical ventilation, neuroinfections, new motor deficits, and pulmonary complications.

Mean PFS and overall survival (OS) were 25.4 ± 22.6 and 29.9 ± 31.9 months, respectively. Postoperative functional assessments clustered most patients within MMCS grades 2-3 and mRS scores between 1 and 3, reflecting moderate disability and partial independence. A detailed breakdown of surgical extent, pathology, clinical improvement, and survival outcomes is provided in Table 2.

**Table 2:** Postoperative clinical, histopathological, and survival outcomes in adult patients with spinal ependymoma Postoperative outcomes including extent of resection, histopathological classification, recurrence rate, clinical improvement, adjuvant therapy, complications, survival times, and functional status evaluated by McCormick and modified Rankin scales in a cohort of adults with spinal ependymoma (n = 56). Data are presented as mean ± standard deviation (SD) for continuous variables and as number (percentage) for categorical variables.

Variable	Value
Extent of Resection, n (%)	
Subtotal resection	20 (36.4%)
Gross total resection (GTR)	35 (63.6%)
Histopathology, n (%)	
Subependymoma	3 (5.4%)
Myxopapillary ependymoma	16 (29.1%)
Classic ependymoma	29 (52.7%)
Anaplastic ependymoma	7 (12.7%)
WHO Grade, n (%)	
Grade 1	3 (5.4%)
Grade 2	45 (81.8%)
Grade 3	7 (12.7%)
Recurrence, n (%)	15 (27.3%)
Number of surgical procedures (mean ± SD)	1.16 ± 0.42
Postsurgical clinical improvement, n (%)	
Pain	22 (40.0%)
Sensory disturbance	18 (32.7%)
Spasticity	3 (5.4%)
Weakness	42 (76.4%)
Urinary incontinence	9 (16.4%)
Bowel dysfunction	6 (10.9%)
Adjuvant therapy, n (%)	
Fractionated radiotherapy	18 (32.7%)
Total radiation dose (Gy, mean ± SD)	52.92 ± 5.46
Lumbar puncture procedures	11 (20.0%)
Number of cells in CSF (mean ± SD)	0.76 ± 3.14
Chemotherapy	1 (1.8%)
Metastatic disease	0 (0.0%)
Postsurgical complications, n (%)	
Neuroinfection	2 (3.6%)
Quadriparesis	1 (1.8%)
Urinary incontinence	3 (5.4%)
Upper extremity pain	1 (1.8%)
Hemodynamic instability	2 (3.6%)
Mechanical ventilation	2 (3.6%)
Lower extremity weakness	2 (3.6%)
Pneumonia	2 (3.6%)
Tracheostomy	1 (1.8%)
Radicular pain	1 (1.8%)
Fecal incontinence	1 (1.8%)
Postsurgical paresthesia	1 (1.8%)
Survival outcomes (months, mean ± SD)	
Progression-free survival	25.37 ± 22.61
Overall survival	29.9 ± 31.91
Functional scales post-surgery, n (%)	
McCormick (%)	
Grade 1	11 (20.0%)
Grade 2	15 (27.3%)
Grade 3	15 (27.3%)
Grade 4	9 (16.4%)
Grade 5	5 (9.1%)
Modified Rankin Scale (mRS) (%)	
Grade 0	4 (7.3%)
Grade 1	13 (23.6%)
Grade 2	11 (20.0%)
Grade 3	10 (18.2%)
Grade 4	6 (10.9%)
Grade 5	10 (18.2%)
Grade 6	1 (1.8%)

Multivariate Cox regression analysis identified thoracic tumor location as a significant predictor of severe postoperative functional limitation (McCormick grades 3-4), with a hazard ratio (HR) of 4.7 (95% CI: 1.25-17.44; p = 0.022). In contrast, GTR and the absence of recurrence were protective factors, with HRs of 0.3 (95% CI: 0.08-0.83; p = 0.023) and 0.2 (95% CI: 0.07-0.75; p = 0.016), respectively. Other variables, including WHO grade, motor deficits, and spasticity, did not reach statistical significance. A detailed summary of the multivariate model is presented in Table 3.

**Table 3:** Multivariate Cox proportional hazards analysis of factors associated with severe functional limitation (McCormick grades 3-4) in postoperative spinal ependymoma patients Multivariate Cox proportional hazards regression analysis evaluating risk factors associated with severe functional limitation—defined as McCormick grades 3-4—after surgery for spinal ependymoma. Results are reported as hazard ratios (HR) with 95% confidence intervals (CI), Wald test statistics, and corresponding p-values. A p-value of <0.05 was considered statistically significant. Variables reaching statistical significance are highlighted in bold.

Variable	Hazard Ratio (HR)	95% Confidence Interval (CI)	p-value
Cervical location	2.1	0.42-10.42	0.373
Thoracic location	4.7	1.25-17.44	0.022
Lumbar location	0.6	0.17-2.12	0.427
Gross total resection	0.3	0.08-0.83	0.023
Recurrence	0.2	0.07-0.75	0.016
WHO Grade 3 (2021)	1.5	0.65-3.50	0.34
Spasticity	1.8	0.67-4.67	0.25
Motor weakness	0.7	0.12-3.72	0.651

Multivariate Cox regression analysis also demonstrated that GTR significantly reduced the risk of tumor recurrence during PFS, with an HR of 0.25 (95% CI: 0.07-0.86; p = 0.028). Other variables, including WHO Grade 3 histology and spinal level, were not independently associated with recurrence. A full summary of the multivariate model is provided in Table 4.

**Table 4:** Multivariate Cox proportional analysis of factors associated with tumor recurrence during progression-free survival in postoperative spinal ependymoma patients Multivariate Cox proportional hazards regression analysis was performed to identify factors associated with tumor recurrence during progression-free survival in patients with spinal ependymoma following surgery. Results are presented as hazard ratios (HR) with 95% confidence intervals (CI), Wald test statistics, and corresponding p-values. Gross total resection was significantly associated with a reduced risk of recurrence (p < 0.05) and is highlighted in bold.

Variable	Hazard Ratio (HR)	95% Confidence Interval (CI)	p-value
WHO Grade III (2021)	1.9	0.67-5.22	0.233
Cervical location	1.6	0.18-15.19	0.663
Thoracic location	2.5	0.37-16.56	0.352
Lumbar location	0.97	0.18-5.24	0.97
Gross total resection	0.25	0.07-0.86	0.028

**Figure 4:**
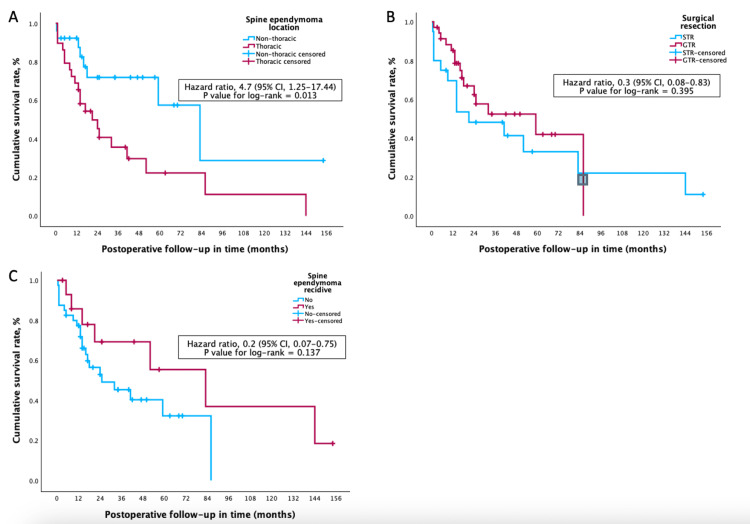
Kaplan-Meier curves depicting progression-free survival (PFS) in patients with spinal ependymoma Kaplan-Meier survival analyses illustrating progression-free survival (PFS) stratified by key clinical variables: (A) PFS according to tumor spinal location (cervical, thoracic, lumbar); (B) PFS based on extent of surgical resection (gross total resection vs. subtotal resection); and (C) PFS relative to tumor recurrence status. These survival curves demonstrate significant differences in PFS associated with the extent of surgical resection and tumor recurrence, underscoring their prognostic importance.

Kaplan-Meier analysis demonstrated an overall PFS rate of 62.1% at 28 months after surgery. Patients who underwent GTR had significantly higher PFS rates, reaching 82.6% at 23 months, compared to 41.9% at 14 months in those who had subtotal resection. These findings are illustrated in Figure 4B, which shows PFS stratified by extent of resection. The complete Kaplan-Meier curves (Figure 4) also explore PFS differences by spinal level (4A) and recurrence status (4C), highlighting the prognostic relevance of surgical extent and disease control.

Consistently, multivariate Cox regression analysis confirmed that GTR was independently associated with a reduced risk of tumor recurrence (HR: 0.25; 95% CI: 0.07-0.86; p = 0.028), as detailed in Table 4.

**Figure 5:**
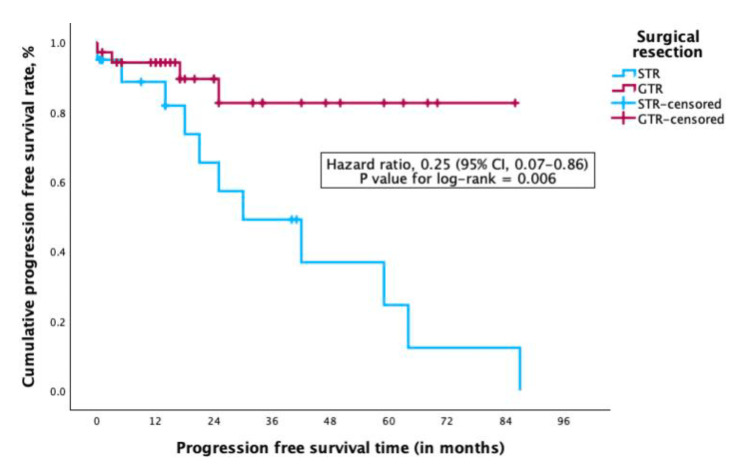
Kaplan-Meier curve comparing progression-free survival (PFS) in patients with and without syringomyelia Kaplan-Meier survival analysis comparing progression-free survival (PFS) between spinal ependymoma patients presenting with syringomyelia and those without. The survival curve demonstrates a trend toward lower PFS in patients with syringomyelia, highlighting its potential impact on disease progression.

Kaplan-Meier analysis showed that patients with syringomyelia exhibited lower PFS, reaching 55.8% at 40 months, compared to 76.9% in those without syringomyelia (Figure 5).

## Discussion

Spinal ependymomas are the most frequent intradural tumors in adults, often manifesting with a wide array of symptoms primarily due to direct spinal cord compression [4-6]. Our study adds valuable data by characterizing clinical, radiological, surgical, and outcome variables in a well-defined adult cohort treated at a national reference center.

Although most published series originate from North America, Europe, or East Asia, Latin American populations remain underrepresented in the literature on spinal ependymomas. Additionally, limited access to molecular diagnostics in resource-constrained settings hinders the implementation of the updated WHO classifications in clinical practice.

In line with prior studies, our cohort showed a slight male predominance (54.5%), with most patients presenting in the fourth decade of life [7]. While some reports have associated ependymomas with neurofibromatosis type 2 [8-10], no such cases were identified in our series.

According to the 2016 WHO classification - which we applied due to the absence of molecular profiling - classic ependymoma (52.7%) and myxopapillary ependymoma (29.1%) were the most prevalent histological subtypes, collectively accounting for over 80% of cases. This distribution is consistent with global data. Although the 2021 WHO update emphasizes molecular profiling for refined classification [11-15], such analyses (e.g., ZFTA, YAP1 fusion status) were not feasible in our setting. Future integration of methylation or fusion-based data may further improve prognostic stratification.

Cervical localization is traditionally considered the most common, except for myxopapillary tumors that predominate in the lumbosacral region [16,17]. Our cohort, however, revealed near-equal involvement of the cervical (54.5%) and thoracic (52.7%) regions, likely reflecting the extensive tumor length (mean: 4.2 vertebral segments) [18,19]. This finding aligns with the broader distribution reported by Davidson et al. [11].

Most patients presented subacutely with sensory disturbances (92.7%), motor weakness (90.9%), and pain (78%). Autonomic dysfunction occurred in nearly one-third of cases, consistent with prior series [5,11]. Notably, complete medullary syndrome (32.7%) was the most frequent neurological pattern, followed by central and cauda equina syndromes (each 9.1%), reflecting the severity of cord compromise.

Baseline neurological impairment is a key determinant of surgical outcome. The prevalence of autonomic dysfunction and subacute presentations suggests substantial cord damage by the time of diagnosis, limiting potential for full recovery. These deficits are notoriously difficult to reverse and are associated with long-term morbidity [20,21].

To comprehensively assess outcomes, we employed three validated scales: the MMCS, mRS, and SS. This multidimensional approach enhances robustness by combining tools for functional grading (MMCS), global disability (mRS), and prognostic stratification (SS) [3]. MMCS and mRS are widely used in spinal and stroke literature, respectively, while the SS - validated by Özkan et al. [3] - offers a concise yet powerful predictor of postoperative recovery based on motor, sensory, and sphincter function [22].

Before surgery, 32.7% of patients had MMCS grade 3 (gait requiring assistance), and 58.2% had an SS score of 3, which is predictive of poor outcomes in over 70% of cases [2]. Additionally, 67.2% had mRS scores ≥3, indicating substantial disability [23]. Postoperatively, we observed improvement in MMCS scores, including a tenfold increase in grade 1 patients (from 1.8% to 20%), and decreased rates of severe disability. However, nearly half (47.3%) remained within mRS 3-5, and one patient deteriorated to mRS 6, highlighting that motor gains do not always translate into full functional recovery.

Our 28-month PFS of 62.1% aligns with prior reports (60-65%) [24]. GTR was strongly associated with improved PFS (82.6% vs. 41.9%), corroborating previous findings by Kumar et al. [25] and others.

Syringomyelia, commonly associated with intramedullary tumors [26], emerged as an independent factor linked to lower PFS (55.8% vs. 76.9%). This supports data from Kato et al., who identified syrinx formation as a surgical challenge and marker of worse prognosis [27]. Although some authors argue that syringomyelia does not affect long-term survival [28,29], our findings emphasize its clinical relevance.

Multivariate Cox analysis confirmed that GTR significantly reduced recurrence and severe functional deterioration, reinforcing its role as a primary therapeutic goal [30]. Conversely, thoracic tumor location was associated with worse postoperative function, likely due to the anatomical complexity and narrower canal in this region, increasing surgical risk [16,31,32]. While maximal resection is desirable, thoracic tumors often require intraoperative compromises to avoid medullary injury - underscoring the need for individualized surgical planning.

Strengths and limitations

This study’s strengths include a large, well-characterized cohort spanning more than a decade, with comprehensive clinical, radiological, and histopathological data. The use of multiple validated functional scales (MMCS, SS, and mRS) and rigorous statistical methods - including Cox regression and Kaplan-Meier analysis - enhances the robustness and reproducibility of our findings. The detailed surgical descriptions, coupled with systematic imaging follow-up and outcome assessment, contribute to the clinical applicability of the results. Additionally, this study provides important insight into spinal ependymoma management in Latin America, a population underrepresented in the literature.

Several limitations should also be acknowledged. The retrospective design and single-center setting may introduce selection bias, as more complex cases are likely referred to tertiary institutions. While this could affect generalizability, it also reflects real-world referral patterns for rare spinal tumors. The lack of molecular tumor profiling limited the ability to apply the updated 2021 WHO classification; however, histopathological subtypes were rigorously analyzed using available resources. Variability in surgical techniques and the selective use of adjuvant therapies may have influenced outcomes, although all procedures followed a standardized posterior approach.

Functional assessments were performed by multiple evaluators without formal blinding or inter-rater reliability testing, which may introduce observer bias; nevertheless, assessments were conducted by experienced attending neurosurgeons using consensus when needed. Eighteen patients were excluded due to incomplete data, potentially introducing attrition bias; however, inclusion criteria were strictly applied to ensure data quality. Finally, the moderate follow-up period may not fully capture late recurrences, highlighting the need for continued longitudinal surveillance in future studies.

## Conclusions

This study provides new clinical insight into the management of adult spinal ependymomas within an underrepresented Latin American population. We highlight a slight male predominance, a peak incidence in the fourth decade of life, and the predominance of classic and myxopapillary histological subtypes based on the 2016 WHO classification. GTR was significantly associated with improved PFS, confirming its importance as a therapeutic objective. Conversely, the presence of syringomyelia and thoracic tumor location were associated with worse functional outcomes and increased recurrence risk. While many patients experienced postoperative motor improvement, a substantial proportion remained functionally impaired, underscoring the persistence of disability despite neurological recovery.

These findings reinforce existing evidence on the prognostic value of the extent of resection and emphasize the need for early diagnosis and timely surgical intervention. Moreover, they support a risk-adapted, individualized approach to treatment - such as prioritizing aggressive resection in non-thoracic tumors and tailoring follow-up based on anatomical and radiological factors - while recognizing that these recommendations are exploratory and hypothesis-generating. Future studies incorporating molecular classification, longer-term follow-up, and standardized assessments of quality of life are essential to refine prognostic models and further personalize care in patients with spinal ependymomas.
